# Genomic and karyotypic variation in *Drosophila parasitoids* (Hymenoptera, Cynipoidea, Figitidae)

**DOI:** 10.3897/CompCytogen.v5i3.1435

**Published:** 2011-08-24

**Authors:** Vladimir E. Gokhman, J. Spencer Johnston, Chiyedza Small, Roma Rajwani, Shawn J. Hanrahan, Shubha Govind

**Affiliations:** 1 Botanical Garden, Moscow State University, Moscow 119991, Russia; 2 Department of Entomology, Texas A&M University, College Station, TX 77843-2475, USA; 3 The City College of The City University of New York, Biology Department MR526, 138th Street and Convent Avenue, New York, NY 10031, USA; 4 The Graduate Center of The City University of New York, 365 Fifth Avenue, New York, NY 10016, USA

**Keywords:** *Drosophila*, Figitidae, parasitoid, genome size, karyotype

## Abstract

*Drosophila melanogaster* Meigen, 1830 has served as a model insect for over a century. Sequencing of the 11 additional *Drosophila* Fallen, 1823 species marks substantial progress in comparative genomics of this genus. By comparison, practically nothing is known about the genome size or genome sequences of parasitic wasps of *Drosophila*. Here, we present the first comparative analysis of genome size and karyotype structures of *Drosophila* parasitoids of the *Leptopilina* Förster, 1869 and *Ganaspis* Förster, 1869 species. The gametic genome size of *Ganaspis xanthopoda* (Ashmead, 1896) is larger than those of the three *Leptopilina* species studied. The genome sizes of all parasitic wasps studied here are also larger than those known for all *Drosophila* species. Surprisingly, genome sizes of these *Drosophila* parasitoids exceed the average value known for all previously studied Hymenoptera. The haploid chromosome number of both *Leptopilina heterotoma* (Thomson, 1862) and *Leptopilina victoriae* Nordlander, 1980 is ten. A chromosomal fusion appears to have produced a distinct karyotype for *Leptopilina boulardi* (Barbotin, Carton et Keiner-Pillault, 1979)(n = 9), whose genome size is smaller than that of wasps of the *Leptopilina heterotoma* clade. Like *Leptopilina boulardi*, the haploid chromosome number for *Ganaspis xanthopoda* is also nine. Our studies reveal a positive, but non linear, correlation between the genome size and total chromosome length in *Drosophila* parasitoids. These *Drosophila* parasitoids differ widely in their host range, and utilize different infection strategies to overcome host defense. Their comparative genomics, in relation to their exceptionally well-characterized hosts, will prove to be valuable for understanding the molecular basis of the host-parasite arms race and how such mechanisms shape the genetic structures of insectcommunities.

## Introduction

Each species has a characteristic genome size and chromosome number. This information often serves as a starting point for obtaining whole genome sequence. It is also useful for cytological or PCR-based genotyping and comparative genomics. *Drosophila melanogaster* Meigen, 1830 is by far the best-studied insect. Availability of its annotated sequence data (Flybase 2011) is facilitating rapid progress as details of novel gene functions are uncovered and analysis of gene interaction networks and pathways is becoming possible. Sequencing of the *Drosophila melanogaster* genome also provided the baseline for the analysis of eleven additional *Drosophila* Fallen, 1823 species, spurring detailed investigation of the evolution of biological processes (Crosby et al. 2007).

Many species of *Drosophila* serve as hosts to parasitic wasps (Schlenke et al. 2007). In spite of spectacular progress on the model organism itself, practically nothing is known about the genomics or genetics of the parasitic wasps. *Leptopilina* Förster, 1869 and *Ganaspis* Förster, 1869 species (Figitidae) attack larval stages, emerge as free-living adults from the pupal cases of their hosts (Schilthuizen et al. 1998, Melk and Govind 1999, Allemand et al. 2002). *Leptopilina boulardi* (Barbotin, Carton et Keiner-Pillault, 1979) is a specialist parasitoid, while *Leptopilina hete*rotoma (Thomson, 1862) is a generalist; these species exhibit distinct strategies to evade or overcome host defense (Schlenke et al. 2007, Kraaijeveld and Godfray 2009, Lee et al. 2009). *Drosophila*-*Leptopilina* interactions have increasingly become important in understanding innate immunity against natural metazoan parasites and the molecular basis of the arms race between insect host/parasites (Chiu et al. 2006; Kraaijeveld and Godfray 2009, Lee et al. 2009, Paddibhatla et al. 2010).

Karyotypes of only two parasitic wasps attacking *Drosophila* spp., namely, *Leptopilina heterotoma* with n = 10 (Crozier 1975) and *Leptopilina clavipes* (Hartig, 1841) with n = 5 (Pannebakker et al. 2004) have been previously reported. These initial results indicate considerable karyotypic diversity within the *Leptopilina* genus, and related taxa. Here we describe the genome sizes and karyotypes of *Leptopilina* species from the *Leptopilina heterotoma* and *Leptopilina boulardi* clades, as well as that of *Ganaspis xanthopoda* (Ashmead, 1896), and discuss the relationship and significance of these observations.

## Material and methods

Wasps were cultured on the *yw* strain of *Drosophila melanogaster* as described in Sorrentino et al. (2004). Origins of the four larval parasitoids of *Drosophila melanogaster*, namely: *Leptopilina boulardi*, *Leptopilina heterotoma*, *Leptopilina victoriae* Nordlander, 1980 and *Ganaspsis xanthopoda* are given in Table 1.

**Table 1.** Origins, genome sizes, and gross karyotypic data of *Drosophila* parasitoids. Genome size of wasp species correlates with total chromosomal length deduced from karyotypic analysis. The total length of the haploid *Ganaspis xanthopoda* chromosome set differs from both *Leptopilina boulardi* and *Leptopilina heterotoma* at p<0.001, and from *Leptopilina victoriae* at p <0.05; *Leptopilina boulardi* differs from both *Leptopilina heterotoma* and *Leptopilina victoriae* at p<0.001 (T-tests for independent samples).

*Genus, species*	*Locality, strain*	*Genome size, mean±SE (Mb), no. specimens studied*	*Chromosome number, (n) 2n/no. (haploid) diploid specimens studied*	*Total length of haploid set, mean±SE (μm)/no. metaphases studied*	*Reference/note*
*Ganaspis xanthopoda*	New York	971.5±6.7/4	(9)/(2)	87.7±8.3/3	Melk and Govind 1999
*Leptopilina boulardi*	G486	370.0±3.2/5	(9)18/(1)1	Not studied	Sorrentino et al. 2002
*Leptopilina boulardi*	17	362.8±1.7/5	(9)18/(7)4	38.6±3.0/7	Schlenke et al. 2007
*Leptopilina boulardi*	France	366.0±2.2/5	Not studied	Not studied	Lanot et al. 2001
*Leptopilina boulardi*	Average	366.3±2.4/15	(9)18/(8)5	38.6±3.0/7	Pooled data
*Leptopilina heterotoma*	New York	461.9±1.9/6	(10)20/(6)9	58.3±2.1/17	Chiu et al. 2006
*Leptopilina heterotoma*	14	460.0±1.4/5	(10)20/(3)5	Not studied	Schlenke et al. 2007
*Leptopilina heterotoma*	Average	460.9±1.7/11	(10)20/(9)14	58.3±2.1/17	Pooled data
*Leptopilina victoriae*	The Netherlands	520.2±0.8/5	(10)/(3)	63.1±4.5/5	Chiu et al. 2006
*Leptopilina* (genus)	Average	424.7±11.0/31	N/A/(20)19	54.4±2.3/29	Pooled data

Flow cytometric analysis of genome size, based on nuclei isolated from heads of females of three species of *Leptopilina*, and *Ganaspsis xanthopoda* was carried out as described before (Johnston et al. 2004), except that propidium iodide (PI) was added to each sample to a final concentration of 50 µg/ml (not 5µg/ml). Samples were prepared as follows: (A) Each wasp species alone, (B) *Drosophila* alone, and (C) 4-6 replicates of a wasp head and a *Drosophila* head prepared together, with mean genome size estimates and standard errors of those estimates based on the 4-6 co-preparations. DNA amount was determined as the ratio of the mean fluorescence of the sample 2C divided by the mean fluorescence of the *Drosophila* standard, multiplied by the genome size of the standard (1C *Drosophila melanogaster* = 175 Mb, 1C *Drosophila virilis* Sturtevant, 1916 = 333 Mb).

Chromosomal preparations for karyology were obtained from cerebral ganglia of male and female prepupae of parasitic wasps according to the technique used by Imai et al. (1988) with modifications. For an initial assessment of the main karyotypic features of *Leptopilina* spp., metaphase plates from a few preparations of *Leptopilina boulardi* and *Leptopilina heterotoma* were stained with Hoechst 33258 (0.2 µg/ml, Molecular Probes) for five minutes. Images were acquired with a Zeiss Laser 510 Scanning Confocal Microscope and formatted with Zeiss LSM5 software. For detailed karyotype analysis, haploid and diploid mitotic divisions were stained with Giemsa and photographed using Zeiss Axioskop 40 FL optic microscope fitted with an AxioCam MRc camera. Metaphase plates with the best chromosomal morphology were used to obtain karyograms. Chromosomes were classified into four groups (metacentrics, submetacentrics, subtelocentrics and acrocentrics) according to Levan et al. (1964). To obtain karyograms, digital images of metaphase plates were processed with Adobe Photoshop. Measurements of chromosomes were taken using Zeiss AxioVision and then processed with STATISTICA (StatSoft Inc. 1995). Relative lengths of chromosomes (RL) were calculated as percentages of the ratio of a particular chromosome relative to total length of the haploid set. Centromere index (CI) is the percentage of the ratio of length of the short arm relative to total length of the particular chromosome.

## Results

### Genome sizes

**Figure 1.**
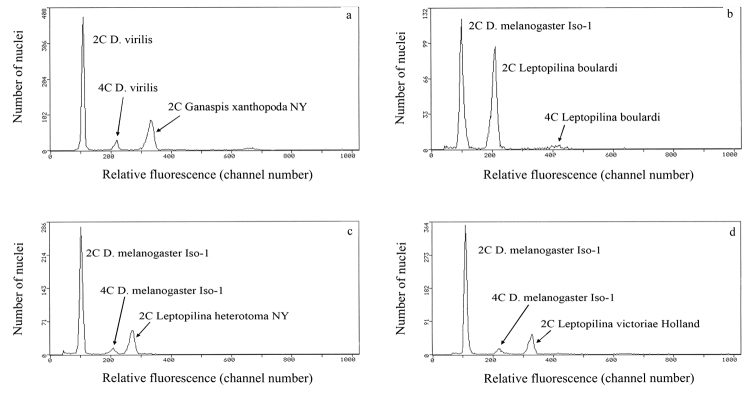
Cytograms showing relative fluorescence and total propidium iodide-stained nuclei of samples and standards to determine genome size. **a** relative fluorescence of PI-stained 2C nuclei from one head of a *Ganaspis xanthopoda* strain NY female co-prepared with 2C and 4C nuclei from one head of a *Drosophila virilis* female standard (1C = 333 Mb) **b–d** relative fluorescence and total PI stained nuclei of co-prepared *Leptopilina* and *Drosophila melanogaster* (1C = 175 Mb) to determine genome size for *Leptopilina boulardi* (panel b), *Leptopilina heterotoma* (panel c), and *Leptopilina victoriae* (panel d). Genome size is calculated as follows: (mean fluorescence channel number of sample 2C peak/mean fluorescence channel number of 2C standard peak) X 1C DNA content of the standard, with the genome size mean and standard error calculated from repeat co-preparations using different individuals of each species.

The results of the study of genome sizes of the *Drosophila* parasitoids show almost no intraspecific variation, yet greater than 2.5-fold interspecific variation (Fig. 1; Table 1). The gametic genome size of *Ganaspis xanthopoda* (1 C = 971 Mb) is larger than that of any of the three *Leptopilina* species (370 Mb < 1C < 520 Mb) studied (Fig. 1). In turn, the genome sizes of all parasitic wasps studied in this paper are also larger than those known for all *Drosophila* species, which range from 1C = 136.5 to 331.5 Mb (Gregory and Johnston 2008).

Our results provide the first information on genome sizes not only of the family Figitidae, but of the superfamiy Cynipoidea as a whole. It is intriguing that the genome sizes of all these parasitoids exceed the average value known for previously studied Hymenoptera, i.e., 360.75 Mb (Tsutsui et al. 2008, Ardila-Garcia et al. 2010), but are fairly close to those of many Chalcidoidea (Tsutsui et al. 2008, Ardila-Garcia et al. 2010), the closest group to cynipoids (see Sharkey 2007).

### Karyotypes

Total lengths of haploid chromosome sets of above mentioned species are given in Table 1. The relative lengths and centromere indices of all chromosomes are given in Table 2.

**Table 2.** Relative lengths (RL) and centromere indices (CI) of *Drosophila* parasitoids. (mean±SE). Strains and numbers of studied metaphase plates are as in Table 1. Centromere indices are: metacentrics: 37.5-50.0; submetacentrics: 25.0-37.5; subtelocentrics: 12.5-25.0; acrocentrics: 0-12.5, according to Levan et al. (1964).

*Species/ chromosome no.*	*Ganaspis xanthopoda*		*Leptopilina boulardi*		*Leptopilina heterotoma*		*Leptopilina victoriae*	
	*RL*	*CI*	*RL*	*CI*	*RL*	*CI*	*RL*	*CI*
1	24.17± 0.77	37.50± 5.34	31.13± 0.81	39.97± 2.98	14.21± 0.32	28.26± 1.42	15.49± 0.21	39.27± 4.55
2	12.85± 0.25	20.50± 1.57	13.06± 0.37	35.13± 2.79	11.89± 0.16	30.03± 1.07	11.63± 0.20	30.46± 4.97
3	11.97± 0.11	20.14± 1.28	11.45± 0.27	29.86± 2.56	11.01± 0.11	28.58± 1.44	11.04± 0.17	32.95± 5.05
4	10.59± 0.32	19.80± 3.50	9.19± 0.17	21.03± 2.99	10.51± 0.82	27.72± 1.16	10.48± 0.19	31.88± 4.34
5	9.35± 0.61	22.25± 3.56	8.54± 0.19	18.66± 4.06	10.02± 0.69	28.90± 1.93	9.53± 0.13	33.69± 5.72
6	8.75± 0.43	35.55± 2.49	7.32± 0.19	17.33± 2.79	9.40± 0.11	33.17± 1.89	9.15± 0.13	31.12± 3.84
7	8.39± 0.15	15.86± 0.39	6.95± 0.15	11.86± 3.68	8.92± 0.11	32.12± 1.96	9.02± 0.11	34.72± 4.95
8	7.81± 0.42	43.48± 1.32	6.42± 0.10	13.91± 4.84	8.48± 0.12	30.16± 1.54	8.69± 0.08	41.12± 2.45
9	6.12± 0.10	1.44± 0.73	5.94± 0.13	8.77± 2.60	8.04± 0.11	28.70± 1.49	8.01± 0.26	34.88± 2.57
10	-	-	-	-	7.52± 0.09	31.93± 1.88	6.96± 0.28	36.19± 1.87

**Figure 2.**
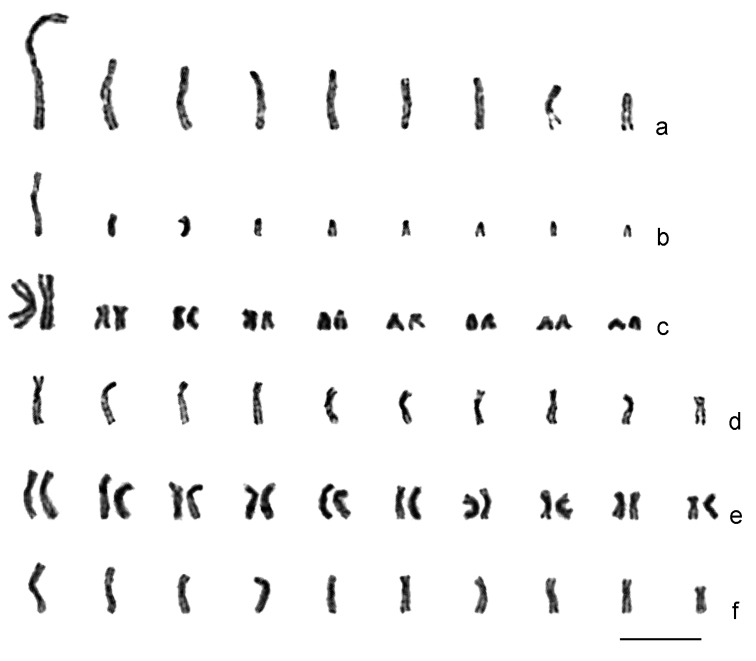
Karyograms of *Drosophila* parasitoids. **a** *Ganaspis xanthopoda*, haploid set **b** *Leptopilina boulardi* (strain 17), haploid set **c** ditto, diploid set **d** *Leptopilina heterotoma* (New York strain), haploid set **e** ditto, diploid set **f** *Leptopilina victoriae*, haploid set. Scale bar 10 μm.

***Ganaspis xanthopoda***. Nine chromosomes were found in the haploid karyotype of this species (n = 9; Fig. 2a). Chromosomes are long relative to *Leptopilina* spp. (see Table 1 and below); most of them are of similar size. However, the first meta- or submetacentric chromosome is about twice as long as the remaining ones. Most other chromosomes are subtelocentric, except for the sixth submetacentric, eighth metacentric, and last acrocentric ones.

**Figure 3.**
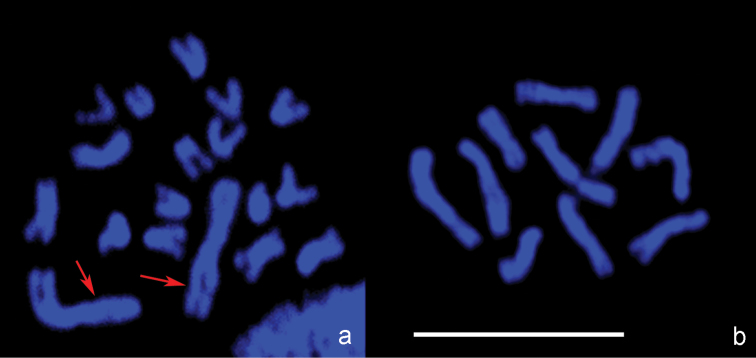
Confocal microscopic images. **a** *Leptopilina boulardi*, diploid metaphase plate **b**- *Leptopilina heterotoma*, haploid metaphase plate. Arrows point to the pair of large metacentric chromosomes in the karyotype of *Leptopilina boulardi* that presumably arose via chromosomal fusion in an ancestral chromosome set with n = 10. Scale bar 10 μm.

***Leptopilina boulardi***. As in *Ganaspis xanthopoda*, n = 9 (and 2n = 18; Figs 2b and c; Fig. 3a). Moreover, the karyotype of *Ganaspis xanthopoda* is superficially similar to that of *Leptopilina boulardi* in that the very large first metacentric chromosome is more than twice as long as the second. However, the length of all remaining *Leptopilina boulardi* chromosomes is roughly half that of the *Ganaspis xanthopoda* chromosomes. Furthermore, chromosomes of the second and third pairs are submetacentric, those of the fourth, fifth, sixth and eighth pairs are subtelocentric, and chromosomes of the seventh and ninth pairs are acrocentric.

***Leptopilina heterotoma***. Consistent with previous observations (Crozier 1975), we found n = 10 and 2n = 20 in this species (Figs 2d and e; Fig. 3b). All chromosomes of the karyotype are submetacentrics that gradually decrease in size.

***Leptopilina victoriae***. This species belongs to the *Leptopilina heterotoma* clade (Allemand et al. 2002), and unsurprisingly, its karyotype is similar to that of *Leptopilina heterotoma*. The haploid karyotype of *Leptopilina victoriae* contains ten submetacentric or metacentric chromosomes (n = 10) of similar size (Fig. 2f). The first chromosome of *Leptopilina victoriae* is significantly longer and the fifth and tenth chromosomes are significantly shorter than the corresponding chromosomes of *Leptopilina heterotoma*. In addition, the centromere position in the first and eighth submetacentric chromosomes is significantly different than observed for the apparently metacentric chromosomes of *Leptopilina victoriae*.

## Discussion

Parasitic wasps make up a significant number of species of all insects (LaSalle and Gauld 1993). However, because of the complete absence of genomic information, the molecular biology and genetics of parasitic wasps of *Drosophila* have lagged behind, even though it is now possible to rapidly sequence genomes of organisms without prior genetic or genomic information.

In a study of genome size of 89 species of bees, wasps, and ants, Ardila-Garcia et al. (2010) hypothesized that genome sizes are constrained by traits associated with parasitism or eusociality. They however found that not all parasitoids have small genomes (Ardila-Garcia et al. 2010). So while it is not altogether surprising that the genomes of the koinobiont parasitoids of *Drosophila* studied here are as large as that of nonparasitic Hymenoptera, it is intriguing that they have such large genomes. Koinobionts keep their host alive; and must develop and emerge before their host is exhausted and dies. Small genomes replicate faster and require fewer resources, which imposes a selection cost on a bloated genome. An antagonist selective force must act on the parasitoid genome. Because of their obligate and intimate relationship with their fly hosts, it is possible that parasitic wasps take up, or share genetic information via transposons. Widespread transfer of genes laterally has recently been documented from *Wolbachia* Hertig, 1936 to insect or nematode genomes (Hotopp et al. 2007). *Wolbachia* has been associated with many parasitic wasps of *Drosophila* (Vavre et al. 2009). Genomic sequence information will reveal if horizontal transmission of transposons, facilitated by the parasitic life style, may have contributed to the large genome size. In this scenario, different transposon types, with rapid turnover in the genome are expected.

**Figure 4.**
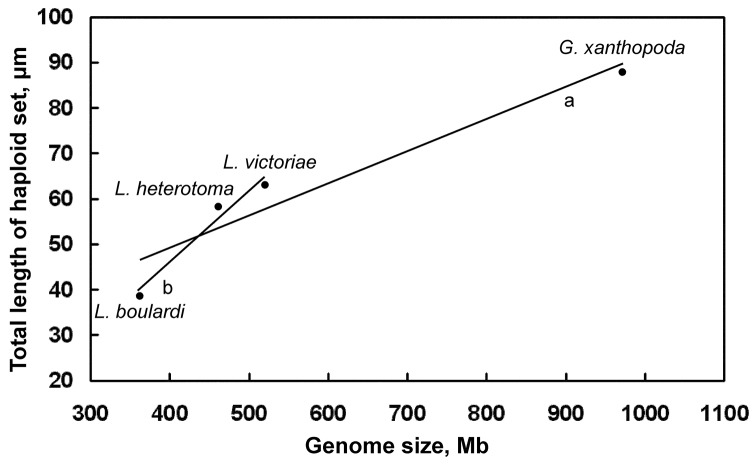
Distribution of genome size/chromosome length of *Drosophila* parasitoids. Mean values are given for each species. Trend lines: **a** for all species combined **b** for *Leptopilina* spp. (i.e. all species excluding *Ganaspis xanthopoda*).

Our karyotypic study provides new insights into the genome structure of *Drosophila* parasitoids. First, the study demonstrates an obvious positive correlation between the genome size and total chromosome length in those parasitic wasps (Table 1; Fig. 4). However, chromosome length in *Ganaspis xanthopoda* increases relatively slower than might be expected from its larger genome size (Fig. 4). This observation suggests that a significant portion of the bloated *Ganaspis* genome is repeat sequence that is highly condensed at metaphase. High copy number of satellite DNA is associated with genome size variation in *Drosophila* species (Bosco et al. 2007) and it is possible that a similar discrepancy in transposon or satellite DNA in the *Ganaspis xanthopoda* genome accounts for smaller than expected increase in chromosome length (Fig. 4).

Second, our study reveals that genome sizes vary independently of the chromosome number in *Drosophila* parasitoids. This may not be surprising if the large metacentric chromosomes of *Leptopilina boulardi* (Fig. 2b; Fig. 3a) and *Ganaspis xanthopoda* (Fig. 2a) have an independent origin via chromosomal fusions. Parallel chromosomal fusions are relatively frequent within various lineages of parasitic Hymenoptera (Gokhman 2004, 2009). In addition to *Ganaspis* and *Leptopilina*, chromosome numbers of *Phaenoglyphis villosa* (Hartig, 1841) (n = 10) and *Callaspidia defonscolombei* Dahlbom, 1842 (n = 11) from the same family have been studied (see Gokhman 2009). This information indicates that n = 10 (or a value close to 10) is likely to be an initial chromosome number for species of the *Leptopilina*/*Ganaspis* clade. If this is true, karyotypes with n = 9 found in *Ganaspis xanthopoda* and *Leptopilina boulardi* as well as that with n = 5 found in *Leptopilina clavipes* are likely to have resulted from chromosomal fusions and are therefore derived from a chromosome set that was probably similar to the karyotypes of *Leptopilina heterotoma* or *Leptopilina victoriae* (see also e.g. Gokhman 2010).

Third, the karyotype provides the scaffold number for future sequencing effort in these insects. When the karyotypic features of the species studied here are superimposed onto their phylogeny (Schilthuizen et al. 1998), clear correspondence is revealed: *Leptopilina heterotoma* and *Leptopilina victoriae* share very similar karyotypes, and are the most closely related species. In contrast, *Leptopilina boulardi* belongs to a distinct clade of the *Leptopilina* genus. Cytogenetic mapping of Expressed Sequence Tags, combined with restriction-site associated DNA (RAD) sequencing (Baird et al. 2008) based on the karyotype would ensure the highest quality genomic assembly, and pave the way for comparative genomics of parasitoid wasps of *Drosophila*. Such comparative genomics will provide insights into the organization of the host and parasitoid genomes and the co-evolution of these insects in nature.
